# Oncogenic mutations of thyroid hormone receptor β

**DOI:** 10.18632/oncotarget.3466

**Published:** 2015-02-28

**Authors:** Jeong Won Park, Li Zhao, Mark Willingham, Sheue-yann Cheng

**Affiliations:** ^1^ Laboratory of Molecular Biology, Center for Cancer Research, National Cancer Institute, National Institutes of Health, Bethesda, MD, USA

**Keywords:** thyroid hormone receptors, oncogenes, protein-protein interactions, signaling transduction, thyroid hormone

## Abstract

The C-terminal frame-shift mutant of the thyroid hormone receptor TRβ1, PV, functions as an oncogene. An important question is whether the oncogenic activity of mutated TRβ1 is uniquely dependent on the PV mutated sequence. Using four C-terminal frame-shift mutants—PV, Mkar, Mdbs, and AM—we examined that region in the oncogenic actions of TRβ1 mutants. Remarkably, these C-terminal mutants induced similar growth of tumors in mouse xenograft models. Molecular analyses showed that they physically interacted with the p85α regulatory subunit of PI3K similarly in cells. *In vitro* GST-binding assay showed that they bound to the C-terminal Src-homology 2 (CSH2) of p85α with markedly higher avidity. The sustained association of mutants with p85α led to activation of the common PI3K-AKT-ERK/STAT3 signaling to promote cell proliferation and invasion and to inhibit apoptosis. Thus, these results argue against the oncogenic activity of PV being uniquely dependent on the PV mutated sequence. Rather, these four mutants could favor a C-terminal conformation that interacted with the CSH2 domain of p85α to initiate activation of PI3K to relay downstream signaling to promote tumorigenesis. Thus, we propose that the mutated C-terminal region of TRβ1 could function as an “onco-domain” and TRβ1 is a potential therapeutic target.

## INTRODUCTION

Thyroid hormone receptors (TRs) are ligand-dependent transcription factors that mediate the biological activities of the thyroid hormone T3 in growth, differentiation, development, and metabolism. There are two major TR isoforms, α and β, which are differentially expressed during development and in adult tissues [1, 2]. TRs consist of an amino terminal, variable in length and sequences (A/B domain), a central DNA binding domain, and a C-terminal T3-binding domain (DE domain). TRs regulate the transcription of their target genes by interaction with specific DNA sequences known as thyroid hormone response elements (TREs). The transcription of TRs is further modulated by recruitment of a host of co-regulatory proteins. In the presence of T3, the T3-bound TR under goes structural changes that result in the release of co-repressors, thus allowing recruitment of nuclear receptor coactivators to facilitate transcription activation [3, 4].

The critical role of TRβ in mediating the biological activities of T3 is clearly evident in that mutations of TRβ cause resistance to thyroid hormone (RTH) [5]. Patients generally have elevated thyroid hormones accompanied by non suppressible thyroid stimulating hormone (TSH). However, the target tissues of patients exhibit reduced sensitivity to thyroid hormone, leading to reduced linear growth, impaired hearing, delayed bone development, and attention deficit disorder [5]. While a few patients with homozygous mutations of the *THRB* gene have been reported [6, 7], whether these patients with two mutated *THRB* alleles also have diseases besides RTH is unknown.

The availability of a mutant mouse harboring a potent negative dominant mutant, TRβPV (*Thrb^PV^* mice) has allowed us to address this question [8]. TRβPV, which was identified in an RTH patient, has a frameshift mutation in the carboxyl-terminal 14 amino acids [9]. As a result of this mutation, TRβPV has completely lost T3 binding activity and transcription capacity. Similar to RTH patients with a single mutated *THRB* allele, heterozygous *Thrb^PV/+^* mice faithfully reproduce symptoms of decreased sensitivity to thyroid hormones in target tissues [8]. Homozygous *Thrb^PV/PV^* mice exhibit severe RTH similar to that reported for the patients with two mutated *THRB* alleles [6, 7]. Remarkably, *Thrb^PV/PV^* mice spontaneously develop follicular thyroid cancer [10–12]. Extensive characterization of TRβPV molecular actions *in vitro* and *in vivo* clearly demonstrated that TRβPV acts as an oncogene [13, 14].

In line with the findings that a mutated TRβ1 (i.e., PV) is oncogenic, recent studies have presented compelling evidence to show that wild-type TRβ1 could act as a tumor suppressor. The expression of TRβ1 in hepatocarcinoma and breast cancer cells reduces tumor growth, causes partial mesenchymal-to-epithelial cell transition, and has a striking inhibitory effect on invasiveness, extravasation, and metastasis formation in mice [15]. Moreover, fibroblast transformation and tumor formation in nude mice induced by oncogenic *ras* are blocked when TRβ1 is expressed [16]. The tumor suppressor function of TRβ1 was also demonstrated in human follicular thyroid cancer (FTC) cells. Expression of TRβ1 in FTC-133 cells reduces cancer cell proliferation and impedes migration of tumor cells through inhibition of the AKT-mTOR-p70 S6K pathway. Expression of TRβ1 in FTC cells inhibits tumor growth in xenograft models [17]. Recently, we also showed that TRβ1stably expressing in breast cancer MCF-7 cells inhibits estrogen-dependent tumorigenesis via down-regulation of the JAK-STAT-cyclin D pathways in xenograft models [18].

**Figure 1:**
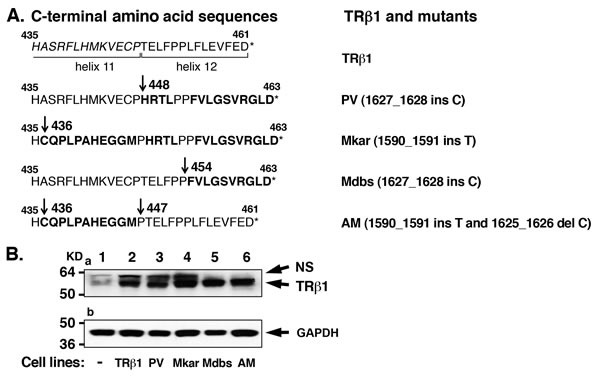
Establishment of cell lines stably expressing TRβ1 and the C-terminal mutants PV, Mkar, Mdbs, and AM in human MDA breast cancer cells A. C-terminal amino acid sequences of TRβ1 and mutants PV, Mkar, Mdbs, and AM. Helix 11 and 12 boundaries are marked. The mutated amino acids are marked in bold. The mutated inserted nucleotides that lead to frame-shift mutations are shown. *Indicates the terminal amino acid. B. TRβ1 and mutants PV, Mkar, Mdbs, and AM were similarly expressed in MDA-MB-468 cells (lanes 2-6), but not in control MDA-MB-468 cells (lane 1). Western blot analysis was carried out as described in Materials and Methods. NS, non-specific bands.

The *apriori* findings raised a fundamental question as to whether the oncogenic action of a TRβ1 mutant is uniquely dependent on the PV mutated sequence or could extend to other C-terminal mutated sequences. The structure of the ligand-binding domain (LBD) of TRβ1 has been determined [19]. The C-terminal helixes 11 and 12 are critically involved in the structural changes of the LBD upon binding of T3 [20]. The frame-shift mutated sequence of PV is located in helix 12 (Figure 1). The availability of two naturally occurring mutants identified in RTH patients [21] has allowed us to evaluate whether other mutations in the C-terminal helix 11 and 12 could also exhibit oncogenic activity. The Mkar mutation has a T insertion at nucleotide 1590_1591 that leads to a frameshift mutation in the terminal 28 amino acids encompassing helix 11 and 12 (Figure 1A). The Mdbs mutation has a C insertion at nucleotide 1643_1644 that leads to a frameshift mutation in the C-terminal 10 amino acids located in helix 12. AM is a mutant that was constructed to combine the part of the mutation from Mkar (amino acids 436-446) and revert the distal amino acid sequence back to that of wild type TRβ1 (amino acids 447-461, located in helix 11) (Figure 1A) [21].

In the present study, we prepared breast cancer cell lines (MDA-MB-468) stably expressing wild type TRβ1 (MDA-TRβ1 cells), PV (MDA-PV cells), Mkar (MDA-Mkar cells), Mdbs (MDA-Mdbs cells), or AM (MDA-AM cells). We chose MDA-MB-468 cells for the studies because they do not express endogenous TRs. We found that cells stably expressing these C-terminal mutants were oncogenic in mouse xenograft models.

These C-terminal mutants physically interact similarly with the p85α regulatory subunit of PI3K to aberrantly activate PI3K-AKT-mTOR and PI3K-ERK-MMP signaling to increase cell proliferation and invasiveness and the PI3K-STAT3-BIM pathway to decrease apoptosis. Thus, we proposed that the helix 11-12 area of mutated TRβ1 could act as an “onco-domain” to drive tumorigenesis.

## RESULTS

### Mutations at the C-terminal helix 11 and 12 lose the tumor suppressor activities of TRβ1

To understand whether mutations in the helix 11 and 12 of TRβ1 are critical to invoke oncogenic activity, we prepared MDA cell lines stably expressing Flag-tagged TRβ1, PV, Mkar, Mdbs, and AM mutations (see mutated sequences in Figure 1A). Mkar and Mdbs mutants were identified in RTH patients [21]. AM containing a partial mutated sequence of Mkar was prepared for comparison (Figure 1A). Figure 1B shows the representative cloned cell lines that had similar protein abundance of these mutants detected by anti-Flag antibodies (Figure 1B, upper panel). GAPDH was used as loading control (Figure 1B, lower panel).

**Figure 2:**
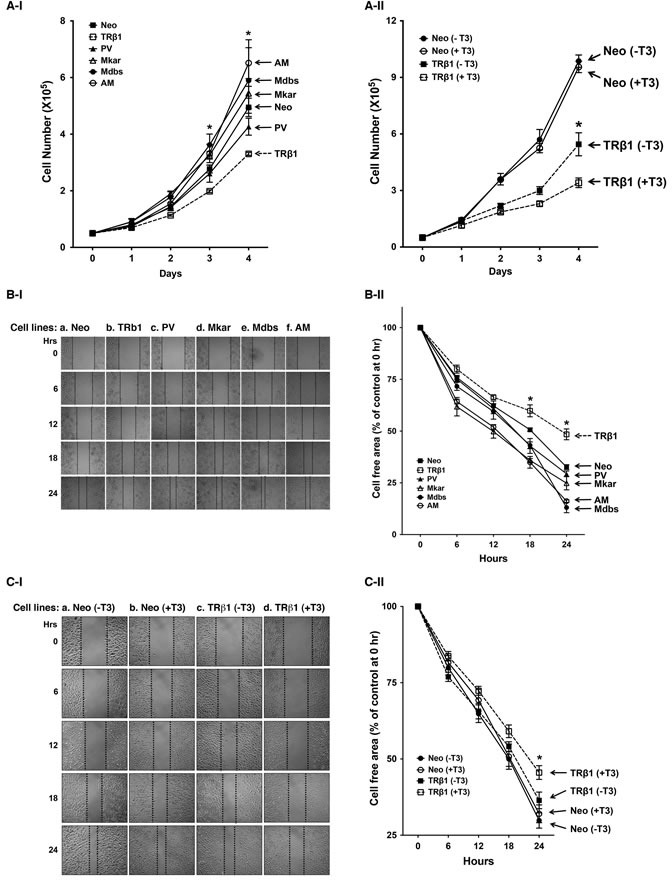
Comparison of rates in cell growth and migration of Neo control cells and MDA-TRβ1, MDA-PV, MDA-Mkar, MDA-Mdbs, and MDA-AM cells A-I. Cell growth was analyzed as described in Materials and Methods. Data are expressed as mean ± standard error (SE) (*n* = 3) and analyzed by one-way ANOVA with Tukey's post-hoc test,* *p* < 0.05. Cell lines are as marked. A-II. Effect of T3 on the proliferation of Neo and MDA-TRβ1 cells. The proliferation assay was carried out as described in Materials and Methods. Data are presented as mean +SE, and analysis between each stable clone was done by using one-way ANOVA with Tukey's post-hoc test, * *p* < 0.05. Cell lines are as marked. B-I. Representative pictures of cell wound healing in Neo cells, MDA-TRβ1 cells, and MDA-C-terminal mutants (PV, Mkar, Mdbs, and AM) cells at 0, 6, 12, 18, and 24 hours. B-II. Cell migration rates determined from results shown in C-I. Data are expressed as mean ± standard error (SE) (*n* = 3), *p < 0.05, MDA-TRβ1 cells vs. Neo control or C-terminal mutants (PV, Mkar, Mdbs, and AM) cells. C-I. Representative pictures of cell wound healing in Neo cells and MDA-TRβ1 in the presence or absence of T3. C-II. Cell migration rates determined from results shown in C-I. Data are expressed as mean ± standard error (SE) (*n* = 3) and the *p*-values are shown.

We have previously shown that TRβ1 acts to inhibit cell proliferation [17, 18, 22]. To evaluate the effects of C-terminal mutations in TRβ1, we compared their proliferation rates with MDA-TRβ1 cells. Figure 2A-I shows that the proliferation rate of MDA-TRβ1 cells was significantly lower than that of control Neo cells, confirming again that TRβ1 acts to inhibit proliferation of MDA cells [22]. Importantly, the proliferation rates of cells stably expressing PV (MDA-PV cells), Mkar (MDA-Mkar cells), Mdbs (MDA-Mdbs cells), or AM (MDA-AM cells) were not significantly different from that of the control Neo cells. These results suggest that the mutations at the C-terminal helix 11 and 12 led to the loss of tumor suppressor activity of TRβ1 by inhibiting cell proliferation.

We also evaluated the effect of T3 on cell proliferation of MDA-Neo and MDA-TRβ1 cells (Figure 2A-II). In MDA-Neo cells that lack TRβ1, no apparent effect of T3 was observed on cell proliferation. However, the proliferation rate of MDA-TRβ1 cells was significantly faster in the absence of T3 than in the presence of T3, indicating that unliganded TRβ1 exhibited oncogenic activity.

We further evaluated whether mutations in the C-terminal helix 11 and 12 of TRβ1 altered the cell migration. Figure 2B-I shows that MDA-TRβ1 cells had a slower rate of migration than the control Neo cells (compare column b vs. a). However, the migration rates of MDA-PV (column c), MDA-Mkar (column d), MDA-Mdbs (column e), and MDA-AM cells (column f) were similar to that of control Neo cells (column a). These findings are evident from the quantitative analyses of the migration rates (Figure 2B-II). The migration rate of MDA-TRβ1 cells was significantly slower than that of control Neo cells. The migration rates of cells stably expressing C-terminal mutants were not significantly different from that of the control Neo cells. Taken together, these results indicate that mutations in the C-terminal helix 11 and 12 led to the loss of functions of TRβ1 in the inhibition of cell proliferation and migration. Moreover, these findings suggest that the C-terminal mutants of TRβ1 could gain oncogenic activities.

We also evaluated the effect of T3 on the cell migration of MDA-Neo and MDA-TRβ1 cells. Figure 2C-Ia and Ib show that no apparent effect of T3 was observed on the cell migration of MDA-Neo cells. Interestingly the migration rate of MDA-TRβ1 in the absence of T3 was similar to that of MDA-Neo cells (Figure 2C-II). However, the migration rate of MDA-TRβ1 cells the presence of T3 was significantly slower than that of MDA-TRβ1 cells without T3 (Figure 2C-II). These results indicate that unliganded TRβ1 exhibited oncogenic activity by promoting cell migration.

### C-terminal mutants of TRβ1 are oncogenic in the mouse xenograft model

**Figure 3:**
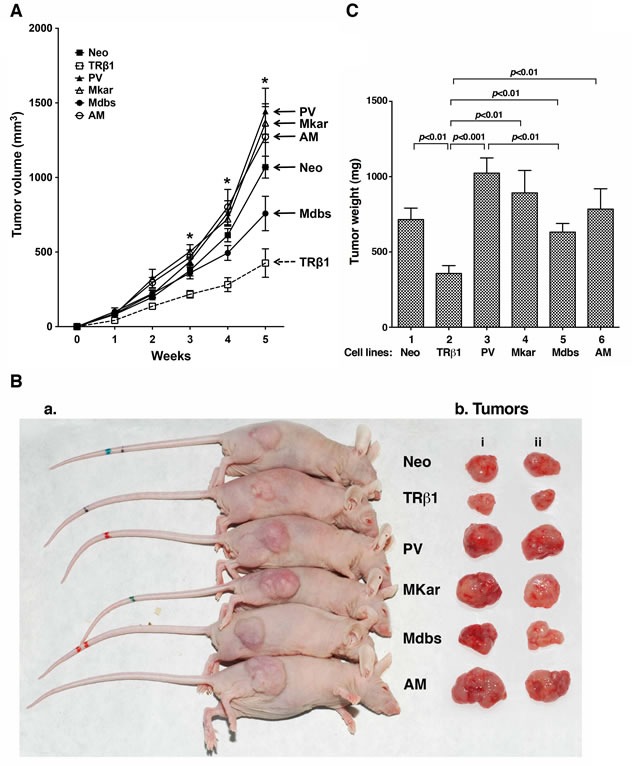
Comparison of tumor growth rates derived from injection of MDA-TRβ1, MDA-PV, MDA-Mkar, MDA-Mdbs, MDA-AM, and Neo control cells A. Equal numbers of cells were inoculated onto the right flank of mice 6-week-old female athymic NCr-nu/nu mice. Tumor sizes were measured weekly and the rates of tumor growth were compared. *p < 0.05. B. Representative pictures of tumors bearing mice (B-a) and dissected tumors (“i and ii” represent duplicates, B-b). C. Tumors were dissected at the endpoint and the weight was determined. The data are expressed as mean ± SE (*n* = 6) and the *p*-values are shown.

We have recently shown that TRβ1 acts as a tumor suppressor to inhibit the induction of MDA tumor growth in mouse xenograft models. To test whether C-terminal mutants of TRβ1 had lost the tumor suppressor functions *in vivo*, we inoculated MDA-PV, MDA-Mkar, MDA-Mdbs, and MDA-AM cells into athymic mice. Figure 3A shows that the tumor growth rate derived from MDA-TRβ1 (open squares) was clearly markedly slower than that of the control Neo cells (solid squares). The tumor growth rates of cells stably expressing PV, Mkar, Mdbs, or AM were indistinguishable from each other and that of the control Neo cells. Importantly, their rates were faster than that of cells stably expressing TRβ1. Moreover, the tumor size derived from MDA-TRβ1 cells was clearly smaller than that from the control Neo cells and from MDA- PV, MDA-Mkar, MDA-Mdbs, and MDA-AM cells, as shown by the representative examples (Figure 3B-a and 3B-b). Figure 3C shows a quantitative comparison of the tumor sizes developed from injecting Neo, MDA-TRβ1, MDA-PV, MDA-Mkar, MDA-Mdbs, or MDA-AM cells. The tumor size from cells expressing TRβ1 was about 30-50% smaller than that from the Neo control cells (bar 2 vs. bar 1), PV (bar 3), Mkar (bar 4), Mdbs (bar 5), or AM (bar 6). Consistent with the cell-based findings shown above, these *in vivo* results indicate that mutations of the C-terminal helix 11 and 12 led to the loss of tumor suppressor functions of TRβ1.

**Figure 4:**
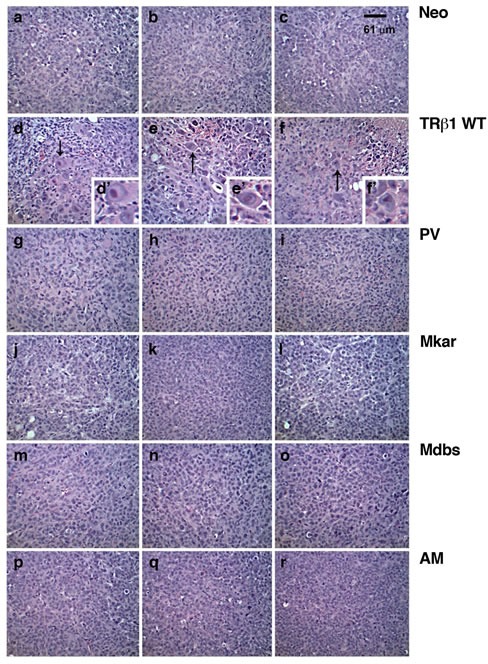
Comparison of histological characteristics in tumors derived from Neo cells, MDA-TRβ1 and MDA-C-terminal mutants (PV, Mkar, Mdbs, and AM) Neo (panels a, b, & c), MDA-TRβ1 (panels d, e, & f), MDA-PV (panels g, h, & i), MDA-Mkar (panels j, k, & l), MDA-Mdbs (panels m, n, & o),and MDA-AM (panels p, q, & r) cells. The magnification was 40X in panels d, e, and f (insets) to indicate large nuclei (arrow) in tumors derived from MDA-TRβ1.

We next compared the morphological features of the xenograft tumors derived from cells expressing TRβ1 or its mutants. Figure 4 shows the morphological features from three representative tumors derived from each cell line. The cells exhibit similar morphology in tumors derived from Neo control cells (panels a, b, & c), MDA-PV (panels g, h, & i), MDA-Mkar (panels j, k, & l), MDA-Mdbs (panels m, n, & o), and MDA-AM cells (panels p, q, & r). The histology in these fast-growing tumors shows small highly proliferative cells with pleomorphic nuclei and prominent nucleoli, numerous mitotic figures, and no significant evidence of necrosis or apoptosis. However, the smaller tumors derived from MDA-TRβ1show scattered cells with large distinct-looking nuclei (marked by arrows in panel d, e, & f, Figure 4; shown clearly in the insets). These unusual-looking large nuclei may reflect consequences of cell cycle arrest, perhaps due to cell fusion and/or failed mitoses, and occasional foci of necrosis and interstitial hemorrhage, all consistent with slow irregular growth and poor cell survival.

**Figure 5:**
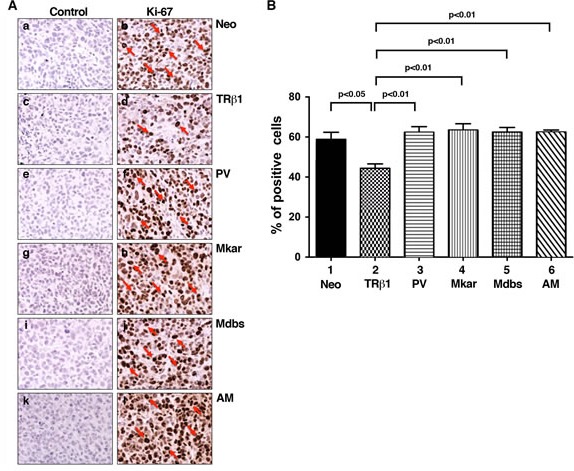
Comparison of cell proliferation by immunohistochemical analysis using the Ki-67 marker in tumor cells derived from Neo control cells, MDA-TRβ1, MDA-PV, MDA-Mkar, MDA-Mdbs, or MDA-AM cells A. Immunohistochemical analysis of protein abundance of the nuclear proliferation marker Ki-67 in tumors. Sections of tumors derived from Neo control cells (panels a & b), MDA-TRβ1cells (panels c & d), MDA-PV cells (panels e & f), MDA-Mkar cells (panels g & h), MDA-Mdbs cells (panels i & j), and MDA-AM cells (panels k & l) were treated with control anti-IgG (panel a, c, e, g, i, & k) or with anti Ki-67 antibodies (panel b, d, f, h, j, & l) as described in Materials and Methods. The Ki-67 positively stained cells are indicated by arrows. B. The Ki-67-positive cells were counted from three different sections and expressed as percentage of Ki-67-positive cells versus total cells examined. The data are expressed as mean ± SE (*n* = 3). The *p*-values are shown.

The slow growth in tumors derived from MDA-TRβ1 cells could be due to decreased cell proliferation and/or increased apoptosis. We, therefore, explored these two possibilities by immunohistochemical analysis. Figure 5A compares the number of cells positively stained by the nuclear proliferation marker Ki-67. Compared with the control Neo tumor cells, tumor cells from the MDA-TRβ1 cell line had markedly fewer cells positively stained with Ki-67 (compare panel d with b). On the other hand, compared with the tumor cells from the MDA-TRβ1 cell line (panel d), tumor cells from MDA-PV (panel f), MDA-Mkar (panel h), MDA-Mdbs (panel j), and MDA-AM (panel l) cell lines all had many more cells positively stained with Ki-67. The number of cells positively stained with Ki-67 were counted and expressed as % of total cell number counted in the entire field (Figure 5B). It is clear that a significant 25% lower number of cells were stained in TRβ1 tumors than in Neo control cells (bar 2 vs. bar 1). However, tumor cells from PV, Mkar, Mdbs, and AM had as many positively stained cells as in the Neo control cells (bars 3, 4, 5, and 6). Panels a, c, e, g, j, and k are the respective negative controls for tumors from Neo control, TRβ, PV, Mkar, Mdbs, and AM cells in which no primary antibodies were used in the experiments. These results indicate that consistent with the cell-based studies shown above, mutations in the C-terminal helix 11 and 12 led to loss of inhibitory activity of cell proliferation by TRβ1 *in vivo*.

**Figure 6:**
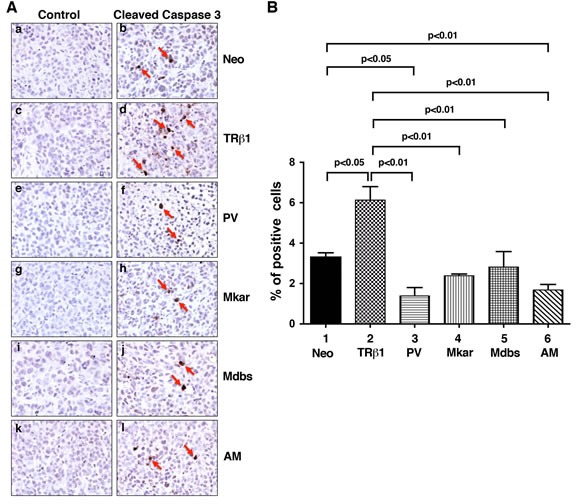
Comparison of apoptosis by immunohistochemical analysis using cleaved caspase 3 as a marker in tumor cells derived from Neo control cells, MDA-TRβ1, MDA-PV, MDA-Mkar, MDA-Mdbs, or MDA-AM cells A. Analysis of the protein expression of cleaved caspase 3 by immunohistochemistry. Sections of tumors derived from Neo control cells (panels a & b), MDA-TRβ1cells (panels c & d), MDA-PV cells (panels e & f), MDA-Mkar cells (panels g & h), MDA-Mdbs cells (panels i & j), and MDA-AM cells (panels k & l) were treated with control anti-IgG (panel a, c, e, g, i, & k) or with anti cleaved caspase 3 antibodies (panel b, d, f, h, j, & l) as described in Materials and Methods. The cleaved caspase 3 positively stained cells are indicated by arrows. B. The cleaved caspase 3-positive cells were counted from three different sections and expressed as percentage of cleaved caspase 3-positive cells versus total cells. The data are expressed as mean ± SE (*n* = 3). The *p*-values are shown.

We further assessed whether the slow growth in tumors derived from MDA-TRβ1 could result from increased apoptosis. Figure 6A compares the number of cells positively stained for the apoptosis marker, cleaved caspase 3, in tumor cells. More cells from TRβ1 tumor cells were stained positively for cleaved caspase 3 than from Neo control tumor (compare panel d to panel b), indicating more tumor cells were undergoing apoptosis in TRβ1 tumors. In contrast, compared with TRβ1 tumor cells, fewer cells from mutant tumors (PV in panel f, Mkar in panel h, Mdbs in panel j, and AM in panel l) were stained positively for cleaved caspase 3. Panels a, c, e, g, i, and k show the negative controls for the respective tumors.

The cells positively stained for cleaved caspase 3 were counted and expressed as % of total cell number counted in the entire field (Figure 6B). It is clear that a significant 45% more cells were stained for cleaved caspase 3 in TRβ1 tumors than in Neo control cells (bar 2 vs. bar 1). The tumors from MDA-PV (bar 3), MDA-Mkar (bar 4), MDA-Mdbs (bar 5), and MDA-AM (bar 6) had significantly lower cell numbers stained for cleaved caspase 3. These results indicate that the increased apoptotic activity in TRβ1 tumors also contributed to decreased tumor growth and that the C-terminal mutations resulted in the loss of apoptotic activity.

### The C-terminal mutants activate PI3K-AKT signaling via interaction with p85α of PI3K

**Figure 7:**
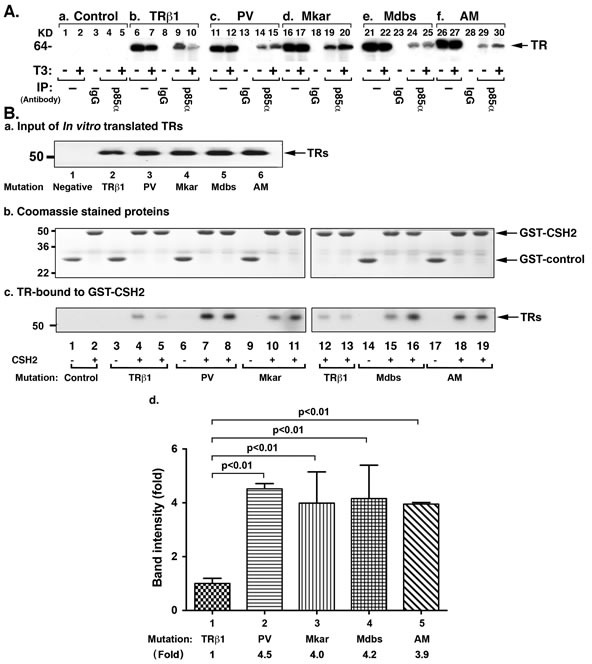
PI3K signal pathway is constitutively activated by C-terminal mutants via protein-protein interaction A. Comparison of co-immunoprecipitated p85α-TRβ1 (lanes 9 & 10), p85α-PV (14 & 15), p85α-Mkar (19 & 20), p85α-Mdbs (24 & 25), and p85α-AM complexes (29 & 30) in the absence (lanes 9, 14, 19, 24, & 29) or presence of T3 (lanes 10, 15, 20, 25, & 30). No co-immunoprecipitated bands were detected in the Neo control cells (lanes 4 & 5). Lanes 6, 7, 11, 12, 16, 17, 21, 22, 26, and 27 show the corresponding input. The IgG controls are shown in lanes 3, 8, 13, 18, 23, and 28. Co-immunoprecipitation was carried out as described in Materials and Methods. B. CSH2 domain protein binds to C-terminal mutants (PV, Mkar, Mdbs, & AM) more avidly than to TRβ1. (a) *In vitro* translated TRβ1 (lane 2) or C-terminal mutants (PV, Mkar, Mdbs, and AM, lanes 2-6, respectively) analyzed by Western blotting using anti-TRβ1 antibody to ensure that equal amounts of receptor proteins were used in the GST-pull down assay. (b) Coomassie blue staining of the SDS-PAGE gel shows that similar amounts of GST-fused proteins were used in the assays (lanes 1-19). (c) Equal amounts of GST-control or GST–CSH2 fusion proteins were each incubated with equal amounts of TRβ1 or C-terminal mutants (PV, Mkar, Mdbs, and AM). After incubation, bound TRβ1 or C-terminal mutants were detected by Western blot using anti-TRβ1 antibody. Lanes 1 and 2 were from the incubation of GST-control or GST-CSH2 domain of p85α with 10 μl of negative control (empty vector), respectively. Lane 3 (GST-control) and lanes 4 and 5 (CSH2 domain of p85α) show the incubation with TRβ1, PV (lanes 6-8), Mkar (lanes 9-11), Mdbs (lanes 14-16), and AM (lanes 17-19), respectively. Duplicates were used for each mutant in the presence of T3. For each mutant, the (d) The band intensities from the binding of TRβ1 and mutants in the presence of T3 (duplicates: lanes 4 & 5 and 12 & 13 for TRβ1) and for mutants (lanes 7 & 8 for PV; lanes 10 &11 for Mkar; lanes 15 & 16 for Mdbs and lanes 18 & 19 for AM) was scanned and quantified by using NIH Image J software.

We have previously shown that PV physically interacts with the p85α regulatory subunit of PI3K, resulting in the activation of PI3K-AKT signaling [23]. We therefore assessed whether other C-terminal mutants-Mkar, Mdbs, and AM-also physically interacted with the p85α subunit of PI3K in cells. We transiently expressed the mutant receptors into MDA cells and compared their interaction patterns with the wild-type TRβ1. We found that while the unliganded TRβ1 was physically associated with p85α (Figure 7A-b, lane 9) in the presence of T3, a markedly decreased TRβ1 was associated with p85α in the presence of T3 (Figure 7A-b, lane 10). Consistent with our previous observations, Figure 7A-c shows that PV physically interacted with p85α independent of T3 (lanes 14 & 15). Remarkably, we found that similar to PV, Mkar (lanes 19 & 20), Mdbs (lanes 24 & 25), and AM (lanes 29 & 30) were also physically associated with p85α independent of T3. We also noted that a similar extent of PV, Mkar, Mdbs, and AM was associated with p85α, whether T3 was present or not. These findings are consistent with the findings that similar to non-T3 binding PV, Mkar, Mdbs, and AM had lost T3 binding activity (data not shown). In line with the finding that patients who express Mkar and Mdbs exhibit RTH, we also confirm that these two mutants, together with AM, displayed dominant negative activity in transcriptional reporter assays (Supplemental Figure A).

p85α consists of a Src-homology 3 (SH3) domain at the amino terminus, a rhoGAP homology domain, and a Src-homology 2 (SH2) domain at the amino terminal side (NSH2) followed by another SH2 domain at the C-terminal end (CSH2) [24]. Previously we showed that the domain in p85α that interacts with the LBD of TRβ1 and PV is CSH2 [23, 25]. We next determined whether similar to PV, Mkar, Mdbs, and AM also interacts with the CSH2 domain of p85α by glutathione S-transferase (GST)-binding assay. An equal amount of *in vitro* transcribed and translated TRβ1 (Figure 7B-a, lane 2) mutant proteins (Figure 7B-a, lanes 3-6) was used to interact with an equal amount of CSH2-conjugated to GST (in duplicates; Figure 7B-b, TRβ1: lanes 4, 5, 12, & 13; lanes 7 & 8, 10 & 11, 15 & 16, 18 & 19 for PV, Mkar, Mdbs, and AM, respectively). As shown in Figure 7B-c, consistent with the cell-based results (Figure 7A) TRβ1 bound to the CSH2 domain (Figure 7B-c, lanes 4, 5, 12, & 13) was clearly weaker than PV (lanes 7 & 8), Mkar (lanes 10 & 11), Mdbs (lanes 15 & 16), and AM (lanes 18 & 19). Lanes 1 and 2 (Figure 7B-c) were the negative controls. In the absence of T3, the binding of TRβ1 and mutants were too weak to observe the signals (lanes 1, 3, 6, 9, 14, 17; Figure 7B-c). We therefore could not compare the binding avidity between TRβ1 and mutants. However, in the presence of T3, clear signals were detected (duplicates: lanes 4 & 5 and 12 & 13 for TRβ1) and for mutants (lanes 7 & 8 for PV; lanes 10 &11 for Mkar; lanes 15 & 16 for Mdbs and lanes 18 & 19 for AM). We therefore compare quantitative data from the determination of the intensities of the bands in the presence of T3 (Figure 7B-d). These results suggest that the C-terminal mutants adopted a conformation favoring a more avid interaction with the CSH2 domain of p85α than with TRβ1.

**Figure 8:**
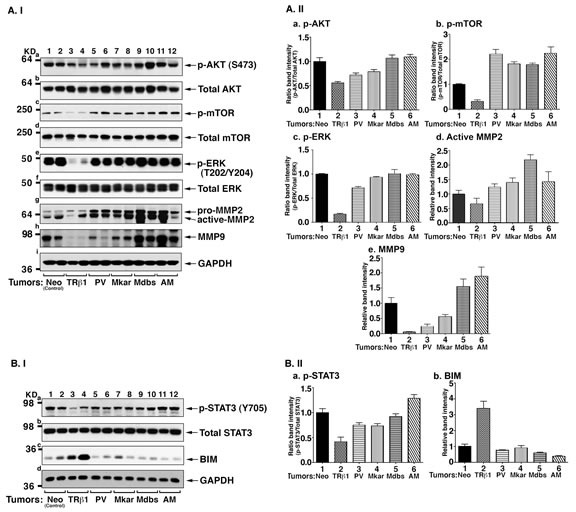
Key downstream regulators of the PI3K signaling pathway are constitutively activated by C-terminal mutants of TRβ1 A. I. Western blot analysis of key regulators, AKT (panels a & b), m-TOR (panels c & d), ERK (panels e & f) and MMP2 (panel g) and MMP9 (panel h) of the PI3K signaling pathway in tumors. Tumors were excised from the injection sites (hind flanks) of athymic nude mice, and the Western blot analysis was carried as described in Materials and Methods. A. II. The band intensities of the protein detected in A-I were quantified and compared. The data are shown as mean ± SE (*n* = 2). B.I. Western blot analysis of key regulators, STAT3 (panels a &b) and BIM (panel c) of the PI3K signaling pathway in tumors. B. II. The band intensities of the protein detected in B-I were quantified and compared. The data are shown as mean ± SE (*n* = 2).

The strong sustained interaction of mutant proteins with the CSH2 domain of p85α shown *in vitro* would predict that the PI3K downstream pathway leads to higher PI3K signaling. We therefore tested this prediction by carrying out Western blot analyses of the downstream key regulators in the PI3K signaling pathway. Figure 8A-I-ashows that reduced phosphorylated AKT (p-AKT) was detected in the tumors induced by MDA-TRβ1cells (lanes 3 & 4), while p-AKT protein abundance in cells expressing mutants (lanes 5-12, two tumors for each mutant expressing cells) was similarly high as that in the control Neo cells (lanes 1 & 2), without significantly affecting the total AKT levels (panel b). Higher levels of p-mTOR, a downstream effector of p-AKT, were detected in all tumors derived from cells expressing mutants (lanes 5-12, panel c) than in tumors derived from MDA-TRβ1 cells (lanes 3 & 4, panel c). No apparent changes were found in total m-TOR (panel d) in all tumors. The ratios of p-AKT/Total AKT and p-mTOR/Total mTOR were quantified from the band intensities to indicate that a higher activation of AKT-mTOR signaling in all tumors derived from cells expressing mutants than from MDA-TRβ1 cells (Figure 8A-II, panels a & b).

Recent studies have shown that the PI3K pathway could cross talk to affect ERK activation [26–28]. Accordingly, we compared the p-ERK protein abundance on tumors derived from MDA-TRβ1 cells and cells expressing mutants. Tumors from MDA-TRβ1 cells had decreased p-ERK protein abundance (Figure 8A-I-e, lanes 3 & 4). In contrast, p-ERK protein abundance in tumors from cells expressing mutants (lanes 5-12) were all elevated as high as that in the Neo control cells (lanes 1 & 2) without affecting the changes in total ERK (panel f). The ratios of p-ERK/Total ERK were quantified from the band intensities to indicate that activation of p-ERK was higher in all tumors derived from cells expressing mutants than from MDA-TRβ1 cells (Figure 8A-II-c). It is known that activation of ERK leads to increases in the expression of matrix metalloproteinase-2 [29]. We therefore assessed the protein abundance of MMP2. Consistent with deactivated p-ERK in tumors from MDA-TRβ1 cells, MMP-2 protein abundance was lower (Figure 8A-I-g, lanes 3 & 4) than the Neo controls (lanes 1 & 2). In contrast, activated p-ERK led to similarly increased abundance of MMP-2 in the tumors derived from cells expressing mutants (lanes 5-12). In addition, we also found that while a reduced MMP-9 was detected in tumors derived from MDA-TRβ1 cells (Figure 8A-I-h, lanes 3 & 4), tumors from cells expressing mutants all had elevated MMP-9 protein abundance (also see Figure 8A-II, panels d & e). These results indicate that the tumor suppressor effects of TRβ1 via the PI3K-AKT/ERK downstream signaling was blocked by mutations in the C-terminal helix 11 and helix 12 as demonstrated for PV, Mkar, Mdbs, and AM.

**Figure 9:**
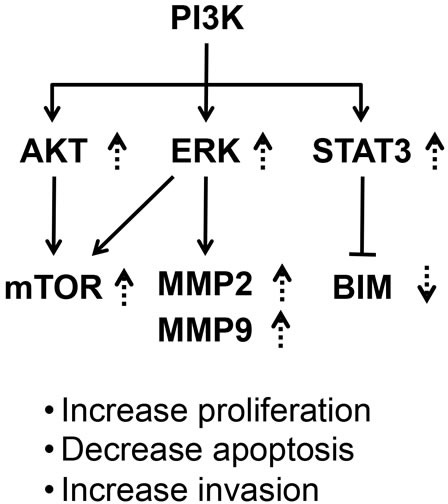
A proposed molecular model to indicate a common activated signaling initiated from activation of PI3K by the C-terminal mutants of TRβ1 Activation of PI3K-mTOR/ERK-MMPs led to cell proliferation and invasion. Activation of PI3K-STAT3-BIM led to decreased apoptosis to promote tumor growth. The broken lines show the changes found in the present studies. The up-arrows show the increases and down-arrows show the decreases.

Recent proteomic data have uncovered an interdependence of PI3K and STAT3 signaling [30]. We therefore further explored whether STAT3 signaling was affected in the tumors derived from TRβ1- and mutant-expressing cells. Interestingly, we found that consistent with the attenuated PI3K-AKT/ERK in tumors derived from MDA-TRβ1 cells, phosphorylated STAT3 at Y705 (p-STAT3Y705) was also decreased (Figure 8B-I-a, lanes 3 & 4). In contrast, activated p-STAT3Y705 in tumors derived from MDA-PV, MDA-Mkar, MDA-Mdbs, and MDA-AM cells (lanes 5-12) was found to be similar to that in control Neo tumors (lanes 1 & 2). No apparent changes were observed in the total STAT3 levels in all tumors (Figure 8B-I-b). The ratios of p-STAT3/total STAT3 were quantified from the band intensities to indicate that activation of p-STAT3 was higher in all tumors derived from mutants than from TRβ1-expressing cells (Figure 8B-II-a). One of the downstream effectors of STAT3 is BIM, a proapoptotic protein [31]. BIM was increased in tumors derived from MDA-TRβ1 cells (Figure 8B-I-c, lanes 3 & 4), but was markedly decreased in tumors derived from cells expressing mutants (lanes 5-12). The quantitative data shown in Figure 8B-II-b demonstrated that BIM was elevated 3.3-fold higher than that in the tumors from the four mutant-expressing cells. These changes were consistent with the increased apoptotic activity as shown by the findings that more cleaved caspase 3 was detected in the nuclear compartment of MDA-TRβ1 cells, but much less in tumors from cells expressing mutants (see Figure 6). Taken together, our results indicate that TRβ1 acts as a tumor suppressor via attenuation of PI3K-AKT/ERK/STAT3 signaling to reduce tumor growth by decreasing cell proliferation and increasing apoptosis, and by impeding tumor cell invasion. In contrast, the C-terminal mutants, PV, Mkar, Mdbs, and AM, have lost these activities, thereby functioning as oncogenes (see Figure 9).

## DISCUSSION

We previously showed that TRβ1 could function as a tumor suppressor in human thyroid cancer cells [17] and breast cancer MCF cells [18]. In the present studies, we further showed that TRβ1 could also act as a tumor suppressor in another breast cancer cell line, MDA cells. Previously, TRβ1 was also shown to suppress tumor invasiveness and metastasis in human liver hepatocarcinoma cells [15]. These cancer cells do not express TRβ1. When TRβ1 was exogenously introduced to express in these cancer cells, the common phenotypes exhibited by these cancer cells were inhibited cell proliferation, impeded cell migration, and suppressed tumor growth in xenograft models. The molecular pathways elucidated that lead to these attenuated cancer phenotypes could differ depending on the cellular content. This is exemplified by the findings in the two lines of breast cancer cells we studied. In MCF cells, which express the estrogen receptor, the tumor suppressor effect of TRβ1 is estradiol-dependent [18]. In MDA cells, which do not express estrogen receptors, the tumor suppressor effect of TRβ1 was observed but is estradiol-independent. In spite of these cell-type differences that affect how TRβ1 could function as a tumor suppressor, one conclusion that we could reach from the aforementioned studies is that these cancer cells have evolved mechanisms to silence the expression of the *Thrb* gene to gain advantages in cell proliferation, migration, and invasion.

Several mechanisms by which the silencing of the *Thrb* gene expression could occur have been proposed. One is via the silencing of the *THRB* expression by hypermethylation on the promoter region. This silencing mechanism has been shown to occur in breast, lung, colon, acute lymphoblastic leukemia, and thyroid cancers [32–37]. Recent studies have provided evidence that the expression of the *THRB* gene could also be repressed through a microRNA regulatory mechanism in papillary thyroid carcinoma [38]. In view of the critical function of TRβ1 as a tumor suppressor, future studies on the elucidation of how the *THRB* gene is silenced in cancer cells could provide additional insights for understanding the important role of TRβ1 in cancer development and progression.

TRβ1 can also lose tumor suppressor functions by mutations. Compelling evidence from cell-based studies as well as mouse models has shown that the potent dominant negative PV mutant is oncogenic. PV could act via nuclear genomic actions to suppress the transcription activity of tumor suppressors such as the peroxisome proliferator-activated receptor γ (PPARγ) [39, 40]. One mechanism by which PV acts to suppress the expression of PPARγ is via the dominant negative action of PV. PV binds to the peroxisome proliferator response element (PPRE) as homodimers and heterodimers with PPARγ or the retinoid X receptor (RXR), thereby competing with PPARγ for binding to PPRE and for sequestering RXR. PPRE-bound PV recruits the nuclear receptor corepressor 1 (NCOR1) to repress the PPARγ-mediated transcriptional activity [39]. Thus, the oncogenic activity of PV can be mediated via dominant negative activity of PV on transcription activity of tumor suppressors. In addition, PV could also act via extranuclear sites-initiated actions to aberrantly activate oncogenic signaling to promote cancer development and progression [13, 14]. However, the fundamental question is whether the oncogenic activity of PV is imparted by the unique mutated PV sequence per se or results from mutations in the helix 11-12 regions of the ligand-binding domain.

Remarkably, the present studies showed that in addition to PV, other C-terminal mutations in the helix 11-12 regions exhibited similar oncogenic actions from losing the tumor suppressor functions of TRβ1 in growth inhibition and impeding cell invasiveness. The morphology of tumor cells derived from cells stably expressing PV, Mkar, Mdbs, or AM in mouse xenograft models were indistinguishable (Figure 4). Interestingly, cellular and molecular analyses identified similar alterations in the oncogenic pathways, albeit with some minor variability in the degree of changes (see Figures 2, 3, 5, 6, & 8). These findings argue against the idea that the oncogenic activity is uniquely dependent on a PV mutated sequence, but rather is derived from the mutated sequences present in the four mutants, PV, Mkar, Mdbs, and AM, favoring the formation of an oncogenic conformation.

The notion that the oncogenic activity of the C-terminal TRβ1 mutants had the sequences favoring certain conformation capable of initiating oncogenic events was supported by the observations from protein-protein interaction studies (Figure 7). Cell-based studies showed that in the presence of T3, the majority of TRβ1 was degraded, whereas the mutants were not degraded. These mutants, which did not bind T3, were found to bind p85α more than TRβ1 did. Interestingly, p85α was associated with a similar extent of PV, Mkar, Mdbs, and AM (Figure 7A). Previously we have shown that the CSH2 domain (amino acids 623-706) of p85α is the interaction region with TRβ1 and PV [25]. *In vitro* GST pull down assays showed that association with the CSH2 domain of p85α was 3-fold higher in the mutants than TRβ1 (Figure 7B-d). Remarkably, the CSH2 domain associated with the four mutants with similar avidity (Figure 7B-d). We would expect the similar degree in the interaction of the CSH2 domain of p85α with the four mutants to initiate similar PI3K-AKT downstream signaling. Indeed, detailed molecular analyses using cell-based approach (see Figure 2) and *in vivo* xenograft studies (see Figures 3, 5, 6, & 8) revealed that PV, Mkar, Mdbs, and AM exhibited similar oncogenic activity to promote cell proliferation, decrease apoptosis, and drive tumor growth. Therefore, the oncogenic activity of these four C-terminal mutants resides in the sequences that can form conformation capable of interacting with the CSH2 domain of p85α. PV was the first to be discovered to have a mutated sequence that is oncogenic, but the oncogenic sequence is not unique to PV, as was shown by the present studies that revealed such oncogenic sequences are also present in Mkar, Mdbs, and AM mutants.

At present, it is unclear what the essential oncogenic sequences are. PV, Mkar, and Mdbs share the common “(454)FVLKGSVRGLD(461)”, but this sequence is not present in AM. The mutated sequence in AM, “(436)CQPLPAHEGGMP(447)”, is not present in PV, Mkar, and Mdbs (see Figure 1A), yet the AM mutant exhibited oncogenic activity similar to that of the others. These considerations suggest that though the C-terminal helix 11-12 mutant sequences are not exactly identical, these dissimilar sequences could still fold into tertiary structures to interact avidly with the CSH2 domain of p85α. Identification of the essential residues in the C-terminal mutated sequences by structural analysis would certainly shed new light on how the oncogenic structures were folded and what were the critical amino acids. However, the determination of the C-terminal “oncogenic” structures of these four mutants awaits future studies.

The dominant negative mutants PV, Mkar, and Mdbs were identified in patients with RTH [9, 21]. The present studies show that these RTH mutants could lead to the development of tumors in xenograft mouse models. While the cases are rare, differentiated thyroid cancer in RTH patients has been reported [41–43]. These results suggest that the loss of tumor suppressor functions of TRβ1 due to mutations could potentially be a risk factor for patients beyond RTH. However, the present studies have elucidated signaling pathways by which the C-terminal TRβ1mutants could act as oncogenes. Currently, several inhibitors for mTOR have been approved for treating patients with cancers [44]. Many inhibitors for PI3K-AKT-mTOR and PI3K-ERK/STAT3 are currently in several phases of clinical trials. It is expected that newer, safer, and more effective inhibitors for PI3K-AKT and its downstream effectors will be developed for better treatment in the near future. The findings from the present studies clearly showed that TRβ1 could be a therapeutic target for cancer treatment.

## MATERIALS AND METHODS

### Generation of MDA cells stably expressing TRβ1 or C-terminal mutants (PV, Mkar, Mdbs, and AM)

MDA-MB-468 cells were a generous gift from Ana Aranda (Universidad Autonoma de Madrid, Madrid, Spain). Establishment of MDA-MB-468 cells stably expressing human TRβ1, the C-terminal mutants (PV, Mkar, Mdbs, and AM), or the control gene (Neo) was described previously [18]. The plasmids for expression cDNA for Mkar, Mdbs, and AM were generous gifts from Roy Weiss of the University of Chicago. Briefly, MDA-MB-468 cells were transfected with the expres­sion plasmid containing cDNA encoding 3Flag-TRβ1 (pcDNA3.1-3Flag-TRβ1), 3Flag-PV (pcDNA3.1-3Flag-PV), 3Flag-Mkar (pcDNA3.1-3Flag-Mkar), 3Flag-Mdbs (pcDNA3.1-3Flag-Mdbs), 3Flag-AM (pcDNA3.1-3Flag-AM), or the empty vec­tor containing only the cDNA for the selector marker, the Neo gene. After transfection, cells were selected with 200 μg/ml G418 (Invitrogen, Carlsbad, CA) for 2 weeks. G418-resistant colo­nies expressing TRβ1 and C-terminal mutants were expanded for sub­sequent experiments. The expression of TRβ1 and C-terminal mutant protein was verified by Western blot analysis using monoclonal anti-TRβ (J53) [45] or anti-Flag antibody (Sigma Aldrich. Cat. F3165).

### Cell proliferation assay

The control (Neo), MDA-TRβ1, and MDA-C-terminal mutants (PV, Mkar, Mdbs, and AM) cells (5 × 10^4^cells per well) were plated in 6-well plates (in triplicates) and cultured for 4 days. Cell counts were measured every 24 hours for 4 days using a cell counter (Beckmann Coulter, Indianapolis, IN), as described previously [18].

To evaluate the effects of T3 on cell proliferation, cells were cultured in the medium containing 10% thyroid hormone–deficient bovine serum (Td medium) for 24 hours. Cells were re-seeded in 6-well plates at a density of 5×10^4^ cells/well in Td medium (day 0). Cells were further cultured in the medium with T3 (100 nM) or without T3, and cells were counted each day for 4 days.

### Wound healing assay

Wound healing assay was carried out as previously described [46] with some modifications. The wound was applied with a pipette tip on the confluent cells, and nonattached cells were removed by gently flushing with fresh media. We visualized cell migration with an inverted microscope at ×100 Mag at every 6 hours for 24 hours. The cell migration was determined at the edges of the wound, and the percentage of migration was determined as the ratios between migrated distance and initial distance of the wound under the microscope at ×100 Mag at every 6 hours for 24 hours.

The evaluation of the effects of T3 on cell migration, a similar method as described was used except that cells were cultured Td medium for 24 hours. Cells were then re-seeded in 6-well plates at a density of 5×10^4^ cells/well in Td medium (day 0). Cells were further cultured in the medium with T3 (100 nM) or without T3, and migration distance was determined as described above.

### *In vivo* mouse xenograft study

The protocols for the use and care of the ani­mals in the present studies were approved by the National Cancer Institute Animal Care and Use Committee. Six-week-old female athymic NCr-nu/nu mice were obtained from the NCI-Frederick animal facility. The control MDA cells (Neo) and MDA-TRβ1 or MDA-C-terminal mutants (PV, Mkar, Mdbs, and AM) (5 × 10^6^cells) in 200 μl sus­pension mixed with Matri gel basement membrane matrix (BD Biosciences, cat. 354234) were inoculated subcutaneously into the right flank of mice, similarly as previously described [18]. The tumor size was measured with calipers weekly until it reached ~2 cm in diameter. The mice were then sacrificed and the tumors dissected. The tumor volume was calculated as L × W × H × 0.5236.

### Histopathologic analysis

Xenograft tumors were dissected and fixed in 10% neutral-buffered formalin (Sigma-Aldrich) and subsequently embedded in paraffin. Five-micrometer-thick sections were prepared and stained with hematoxylin and eosin (H&E). Immunohistochemistry was performed on formalin-fixed paraffin tumor sections, as previously described [47]. Primary antibodies used were anti-Ki-67 antibody (dilution 1:300; Thermo Scientific, Fremont, CA; #RB-9043-P0) and anti-cleaved caspase-3 antibodies (1:300 dilution; Cell Signaling, Cat #: 9661). Staining was developed with 3,30 diaminobenzidine (DAB) using the DAB substrate kit for peroxidase (Vector Laboratories, Burlingame, CA, SK-4100). For quantitative analysis Ki-67 or cleaved caspase-3 positive cells were counted by using NIH Image J software version 1.47 (Wayne Rasband, National Institutes of Health, Bethesda, MD).

### Western blot analysis and co-immunoprecipitation assays

The Western blot analysis was carried out as described by Park *et al.* [18]. Anti-TRb1 antibodies (J53; 2 μg /mL) [45] were used. The anti-p-AKT (S473, cat. #9271; 1:500 dilution), total-AKT (cat. #9272; 1:1000 dilution), p-mTOR (S2448, cat. # 2971; 1:500 dilution), total-mTOR (cat. #2972; 1:1000 dilution), p-ERK (T202/Y204, cat. # 9101; 1:1000 dilution), total-ERK (cat. #9102; 1:1000 dilution), p-STAT3 (Y705, cat. # 9131; 1:500 dilution), total-STAT3 (cat. # 9132; 1:1000 dilution), BIM (cat. # 2933; 1:1000 dilution) and GAPDH (cat. #2118; 1:1000 dilution) were purchased from Cell Signaling Technology. Anti-MMP2 (sc-10736; 1:200 dilution), and MMP9 (sc-6841; 1:200 dilution) were purchased from Santa Cruz Biotechnology.

Co-immunoprecipitation of TRβ1 or C-terminal mutants (PV, Mkar, Mdbs, and AM) with p85α was carried as described previously [48]. Briefly, MDA-TRβ1, MDA-C-terminal mutants (PV, Mkar, Mdbs, and AM), and Neo cells were treated in the absence or presence of T3 (100 nM) for 24 hours. Cell lysates (1 mg) were prepared and immunoprecipitated with monoclonal anti-p85α antibody (4 μg) or control rabbit anti-IgG antibodies (4 μg), followed by Western blot analysis using anti-TRβ1antibodies (J53).

### Glutathione S-transferase (GST)-binding assay

Binding of TRβ1 or C-terminal mutants (PV, Mkar, Mdbs, and AM) to GST–CSH2 of p85α was carried out as described in [25] with modifications. *In vitro* translated TRβ1 and C-terminal mutants (PV, Mkar, Mdbs, and AM) were synthesized by using a TNTT7 quick coupled *in vitro* transcription/translation kit (Promega). *E.coli* expressed GST or GST fused p85α-CSH2 protein (~5 μg) was used in each binding reaction. Their concentration was determined by coomassie blue-stained band intensities using bovine serum albumin standards after migration in SDS-PAGE gel. For relative binding affinity studies, identical amounts of *in vitro*-translated TRβ1 and C-terminal mutants (PV, Mkar, Mdbs, and AM) were used. To that purpose, the bound proteins were analyzed by Western blotting using anti-TRβ1 antibody, and the band intensities of TRβ1 or C-terminal mutants (PV, Mkar, Mdbs and AM) were quantified with NIH IMAGE software (Image J 1.47v; Wayne Rashband, NIH).

### Statistical analysis

All data are expressed as mean ± the standard error of the mean (SEM). Significant differences between groups were calculated using Student's *t*-test with the use of GraphPad Prism 6 (GraphPad Software, Inc., San Diego, CA). *p* < 0.05 is considered statistically significant.

## SUPPLEMENTARY MATERIAL


