# Supplementary material for: Oncogenic mutations of thyroid hormone receptor β
Source: Oncotarget. 2015 Feb 28;6(10):8115–31. doi: 10.18632/oncotarget.3466 (PMC4480739; doi:10.18632/oncotarget.3466)
Supplement: Supplementary file 1 [file oncotarget-06-8115-s001.pdf]

Oncogenic mutations of thyroid hormone receptor β

Supplementary Material

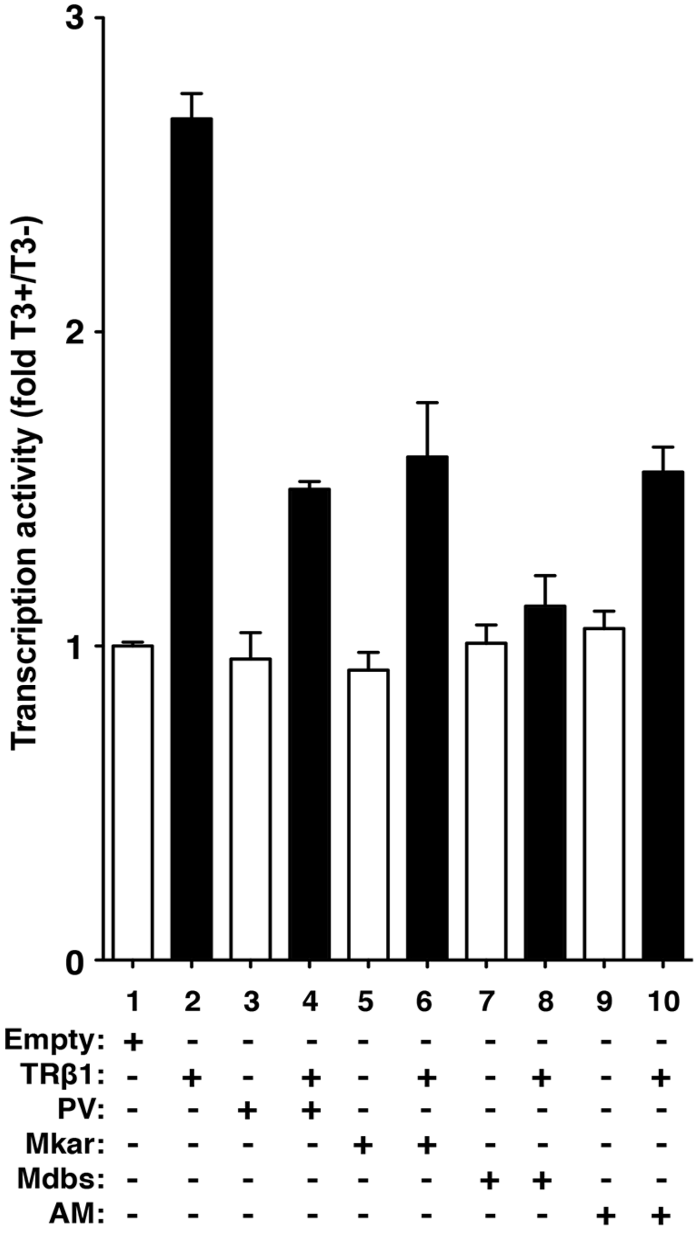

Supplemental Figure A. Similar extent of dominant negative activity in the C-terminal mutants. The expression vectors for TRβ1 (0.2 µg /well), mutants (each 2.0 µg /well) and the Pal-Luc reporter (0.2 µg /well) were transiently transfected to MDA cells in the presence or absence of T3 (100 nM). The lanes are as marked. No transcription activity was observed for mutants (lane 3, 5, 7 and 9). Mutants acted to interfere with the transcriptional activity mediated by TRβ1 (compare lanes 4, 6, 8 and 10 with lane 2).
